# Biochemically Evolving Acute Hepatic Porphyria With Postpartum Progression to Severe Recurrent Neurovisceral Disease

**DOI:** 10.7759/cureus.109789

**Published:** 2026-05-28

**Authors:** Niyas Khalid Ottu Para

**Affiliations:** 1 Internal Medicine, Burjeel Hospital, Abu Dhabi, ARE

**Keywords:** 5-aminolevulinic acid, acute hepatic porphyria, givosiran, hemin therapy, neurovisceral attacks, porphobilinogen (pbg)

## Abstract

Acute hepatic porphyria (AHP) is a group of rare metabolic disorders of hepatic heme biosynthesis characterized by episodic neurovisceral crises resulting from the accumulation of neurotoxic heme precursors, particularly δ-aminolevulinic acid (ALA) and porphobilinogen (PBG). Despite the availability of biochemical testing, diagnosis is frequently delayed because of fluctuating disease expression, non-specific clinical manifestations, normal structural imaging, and challenges related to the timing of biochemical sampling.

We report the case of a 34-year-old female patient with a prolonged six-year history of recurrent unexplained neurovisceral attacks involving severe abdominal pain, nausea, vomiting, anorexia, autonomic instability, weakness, fatigue, dark urine, and progressive neuropathic manifestations. She underwent extensive evaluations across multiple healthcare systems in the United Arab Emirates, India, and Thailand without a unifying diagnosis. AHP was clinically suspected approximately two years before biochemical confirmation because of the highly stereotyped phenotype and reproducible response to 10% dextrose and intravenous hemin despite initially negative ALA and PBG studies. The disease entered complete remission during pregnancy, followed by dramatic postpartum escalation with recurrent hospitalizations, hemin dependence, progressive neurological involvement, autonomic dysfunction, and weight loss. Serial porphyrin fractionation demonstrated persistent and progressive elevation of uroporphyrins and coproporphyrin III before eventual capture of elevated urinary ALA and PBG through strict pre-treatment sampling. Extensive evaluation excluded mitochondrial disease, heavy metal toxicity, Wilson's disease, organic acidemias, fatty-acid oxidation defects, inflammatory abdominal disease, and structural pathology. Whole-genome sequencing was negative for pathogenic AHP variants, highlighting the limitations of genetics when the clinicobiochemical phenotype is compelling. The patient was transitioned to givosiran after developing recurrent hemin-dependent attacks with progressive neurological and autonomic involvement.

This case highlights the evolving biochemical nature of AHP, the diagnostic pitfalls, the limitations of isolated laboratory interpretation, the importance of correct sampling timing, the diagnostic significance of mechanism-directed therapeutic response, the critical role of longitudinal phenotype-driven clinical reasoning in rare metabolic disease, and the transformative role of targeted therapy.

## Introduction

Porphyrias are a heterogeneous group of metabolic disorders caused by disruption of the heme biosynthetic pathway. Heme synthesis occurs through eight enzymatic steps distributed between the mitochondria and cytosol, beginning with condensation of glycine and succinyl-CoA by δ-aminolevulinic acid (ALA) synthase and ending with insertion of iron into protoporphyrin IX by ferrochelatase. The pathway is active in both erythroid tissue and the liver, and clinical phenotypes differ according to the tissue predominantly involved and the level of enzymatic disruption [1,2].

Clinically, porphyrias are broadly classified into acute hepatic porphyrias (AHPs), cutaneous porphyrias, and erythropoietic porphyrias. AHPs include acute intermittent porphyria due to hydroxymethylbilane synthase deficiency, hereditary coproporphyria due to coproporphyrinogen oxidase deficiency, variegate porphyria due to protoporphyrinogen oxidase deficiency, and the rare ALA dehydratase deficiency porphyria. These disorders share a common final pathway involving hepatic ALAS1 upregulation, accumulation of neurotoxic intermediates, and episodic neurovisceral crises [2-4].

The classical acute attack consists of severe abdominal pain, nausea, vomiting, constipation or ileus, tachycardia, hypertension or hypotension, hyponatremia, psychiatric manifestations, peripheral neuropathy, autonomic dysfunction, and dark urine. Despite this recognizable syndrome, AHP is frequently missed because abdominal imaging is often normal, routine laboratory investigations may be unrevealing, and symptoms overlap with functional gastrointestinal disorders, gynecologic disease, autonomic disorders, psychiatric illness, and chronic pain syndromes [3-6].

Importantly, AHP should not be viewed as a static binary biochemical disorder but rather as a dynamic metabolic disease with highly variable penetrance and evolving clinical expression. Disease activity depends not only on the underlying enzymatic defect but also on the interaction between hormonal state, nutritional continuity, metabolic stress, hepatic cytochrome demand, environmental triggers, and cumulative physiological burden. Consequently, some patients may initially demonstrate only intermittent or partially expressed biochemical abnormalities before progressing toward more classical disease phenotypes [1,2,7].

AHPs are rare metabolic disorders, with the prevalence of clinically symptomatic disease estimated at five to 10 cases per million population, although pathogenic variants demonstrate markedly incomplete or low penetrance. Symptomatic disease occurs predominantly in women, typically after puberty and during reproductive years, reflecting the important role of hormonal, metabolic, and environmental triggers in precipitating attacks.

Biochemical diagnosis relies primarily on demonstrating elevated urinary ALA and PBG during an acute attack. However, real-world clinical practice introduces major diagnostic pitfalls. Patients in severe pain frequently receive glucose, opioids, antiemetics, or hemin before urine is collected. Because glucose and hemin suppress hepatic ALAS1 activity, delayed sampling may underestimate or completely miss precursor elevations. Furthermore, fluctuating disease expression, evolving biochemical activity, and variable penetrance may complicate the interpretation of isolated laboratory results [4,5,8].

We report the case of a patient with biochemically evolving AHP characterized by years of fluctuating neurovisceral symptoms, intermittent biochemical abnormalities, pregnancy-related remission, postpartum transformation into severe recurrent disease, delayed biochemical confirmation despite strong clinical suspicion, and early therapeutic response to pathway-directed treatment. This case highlights the importance of longitudinal phenotype recognition, correct biochemical sampling timing, and clinico-biochemical integration in diagnostically evolving metabolic disease.

## Case presentation

A 34-year-old woman had a history of recurrent episodic neurovisceral symptoms involving severe and debilitating abdominal pain, nausea, vomiting, loss of appetite, numbness, paresthesias, tremors, palpitations, tachycardia, extreme fatigue, and weakness for approximately four years before coming under specialist care. During this period, she underwent multiple evaluations in the United Arab Emirates, India, and Thailand. Despite repeated consultations and investigations, no unifying diagnosis was established. Her symptoms were recurrent, disabling, and stereotyped but were repeatedly approached as non-specific abdominal pain.

When she first came under our care approximately two years before biochemical confirmation, her attacks were less frequent than later in the disease course, occurring approximately two times over six months. However, the clinical pattern was already strongly suggestive of AHP. Each episode consisted of severe diffuse abdominal pain associated with nausea, vomiting, loss of appetite, palpitations, tachycardia, hypotension, extreme weakness, and profound fatigue. The attacks were not explained by imaging or routine laboratory testing. Importantly, conventional analgesics and standard emergency treatments provided little benefit, while she repeatedly improved with metabolic treatment, particularly 10% dextrose infusion and, during more severe episodes, hemin infusion. This reproducible treatment response became an important clue suggestive of an underlying inducible hepatic metabolic pathway. To improve chronological clarity in this longitudinally evolving disorder, the major clinical phases, diagnostic milestones, and therapeutic transitions are summarized in Table 1.

**Table 1:** Chronological disease evolution and diagnostic milestones in biochemically evolving acute hepatic porphyria ALA: aminolevulinic acid; PBG: porphobilinogen

Disease phase	Approximate timeline	Clinical pattern	Diagnostic/Therapeutic significance
Early symptomatic phase	Approximately four years before specialist care	Recurrent episodic severe abdominal pain, nausea, vomiting, anorexia, palpitations, tachycardia, hypotension, weakness, fatigue, and disabling stereotyped neurovisceral symptoms; multiple evaluations across the United Arab Emirates, India, and Thailand without a unifying diagnosis	Repeatedly approached as nonspecific abdominal pain despite recurrent stereotyped physiology
First specialist evaluation	Approximately two years before biochemical confirmation	Lower-frequency attacks (approximately two episodes over six months) with a clinical pattern strongly suggestive of acute hepatic porphyria	Early recognition of phenotype despite initially incomplete biochemical confirmation
Early therapeutic responsiveness	During recurrent attacks prior to biochemical confirmation	Limited response to conventional analgesics and standard emergency treatment; repeated improvement with 10% dextrose infusion and, during more severe attacks, hemin therapy	Reproducible response to pathway-directed metabolic therapy supported suspicion of an inducible hepatic heme pathway disorder
Pregnancy-associated phase	During gestation	Marked reduction/remission of neurovisceral attacks	Suggested hormonal and metabolic modulation of disease activity
Postpartum escalation	Following delivery	Progressive increase in attack frequency and severity with recurrent emergency visits, hospitalizations, autonomic instability, neurological symptoms, nutritional decline, and hemin dependence	Transformation into a severe recurrent neurovisceral phenotype
Biochemical evolution	During the longitudinal workup	Persistent elevation of coproporphyrin III and evolving porphyrin abnormalities preceding definitive biochemical confirmation	Suggested evolving heme pathway dysfunction before capture of neurotoxic precursor elevation
Biochemical confirmation	During a symptomatic episode prior to treatment	Elevated urinary δ-ALA and urinary PBG were obtained during active symptoms	Confirmed diagnosis of acute hepatic porphyria
Disease-modifying therapy initiation	Following biochemical confirmation	Givosiran was initiated after recurrent attacks and repeated admissions	Transition from episodic rescue management to mechanism-based preventive therapy
Early follow-up after givosiran	First three months after initiation	Marked reduction in debilitating neurovisceral attacks; only one emergency department visit (during the first month); substantial patient-reported improvement in pain; persistent nausea, anorexia, and limited weight recovery	Early clinical stabilization and major reduction in hospitalization burden despite incomplete symptom resolution

At that stage, urinary ALA and PBG testing did not provide confirmation. Genetic testing for porphyria-associated genes was also unrevealing. Despite this, the pattern of recurrent neurovisceral symptoms, autonomic instability, absence of structural explanation, and reproducible response to dextrose and hemin maintained a high clinical suspicion for AHP.

The patient subsequently became pregnant and was lost to follow-up for approximately one year. During pregnancy, she experienced complete remission of attacks. This was clinically striking because the attacks had been recurrent before pregnancy and later returned after delivery. Approximately nine months postpartum, the disease re-emerged in a much more dramatic and classical form. The attacks became more severe, more frequent, and more disabling. Over approximately four months, she had more than six acute presentations requiring emergency department care and inpatient admissions. At least four episodes required treatment with intravenous hemin infusion, while other attacks required urgent 10% dextrose infusion.

The postpartum attacks were characterized by severe abdominal pain, nausea, vomiting, anorexia, black or dark-colored urine, tachycardia, palpitations, hypotension, profound weakness, and progressive nutritional decline. The urine frequently became markedly dark and occasionally appeared almost black, particularly during severe symptomatic episodes and after standing for prolonged periods. She lost significant weight, with BMI falling from approximately 25 kg/m^2^ to 21 kg/m^2^ over a few months, consistent with systemic burden, reduced intake, and recurrent catabolic stress. Neurological symptoms became more prominent and concerning. She described numbness and paresthesias extending from the abdomen to the lower limbs and subsequently upward toward the upper body and face. These symptoms were accompanied by intense fear, anxiety, autonomic instability, and severe pain.

**Figure 1:**
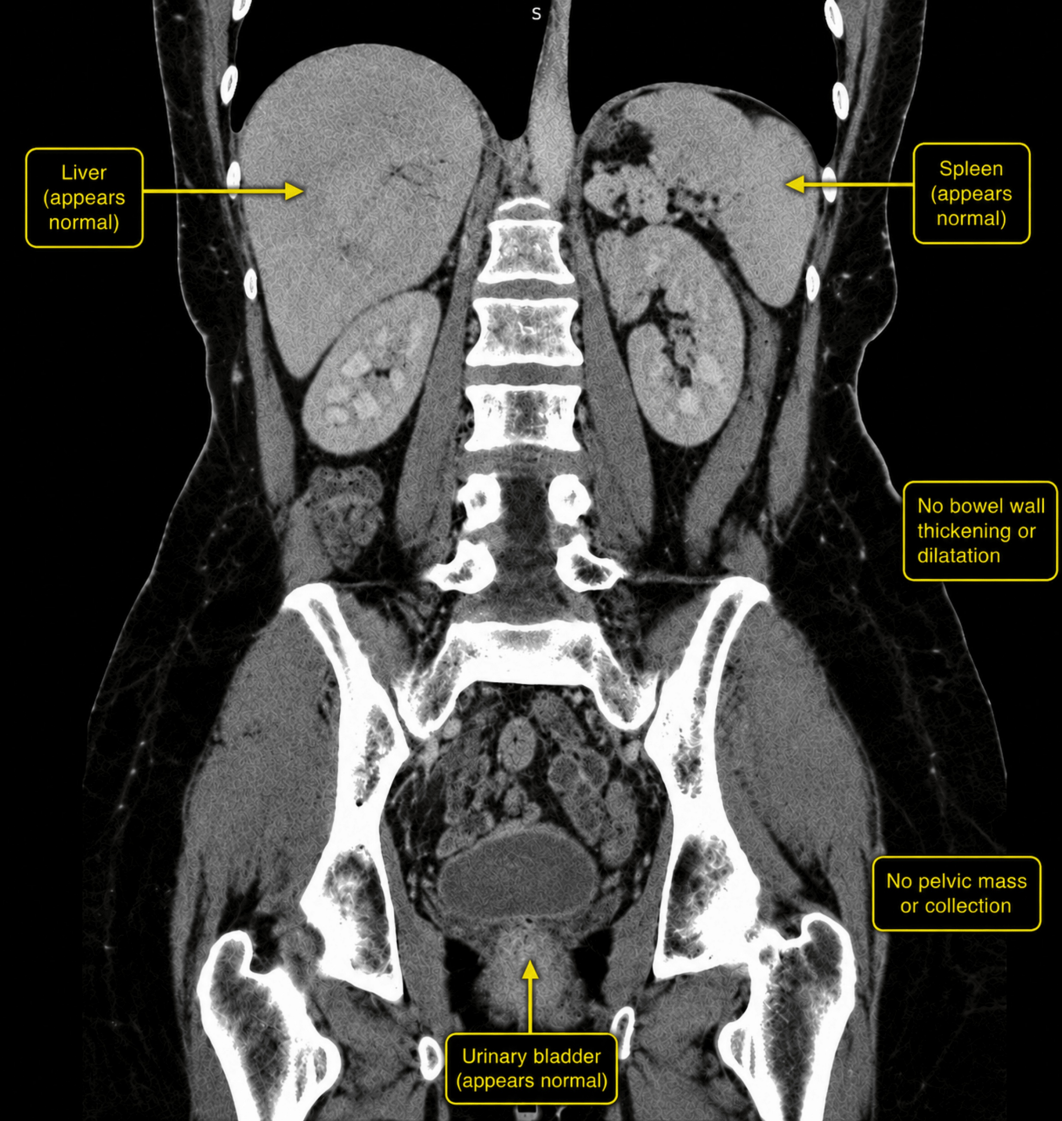
Contrast-enhanced CT of the abdomen and pelvis Contrast-enhanced CT of the abdomen and pelvis performed during recurrent severe neurovisceral attacks demonstrating the absence of structural intra-abdominal pathology despite severe neurovisceral symptoms.

**Figure 2:**
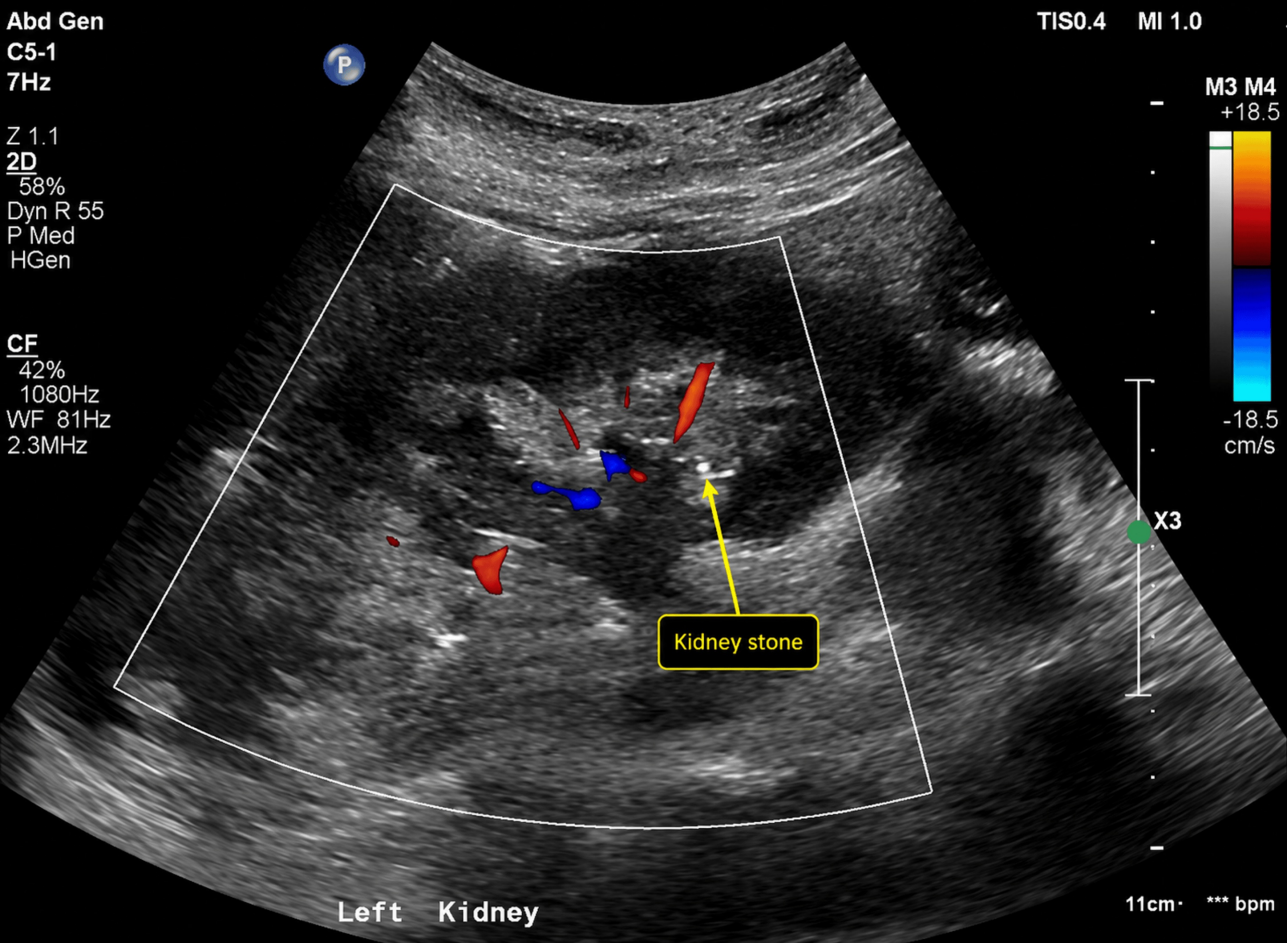
Ultrasound of the abdomen Ultrasound of the left kidney demonstrating a small non-obstructive renal calculus (arrow) without hydronephrosis or significant obstructive uropathy. The finding was considered incidental and insufficient to explain the recurrent severe neurovisceral attacks.

**Figure 3:**
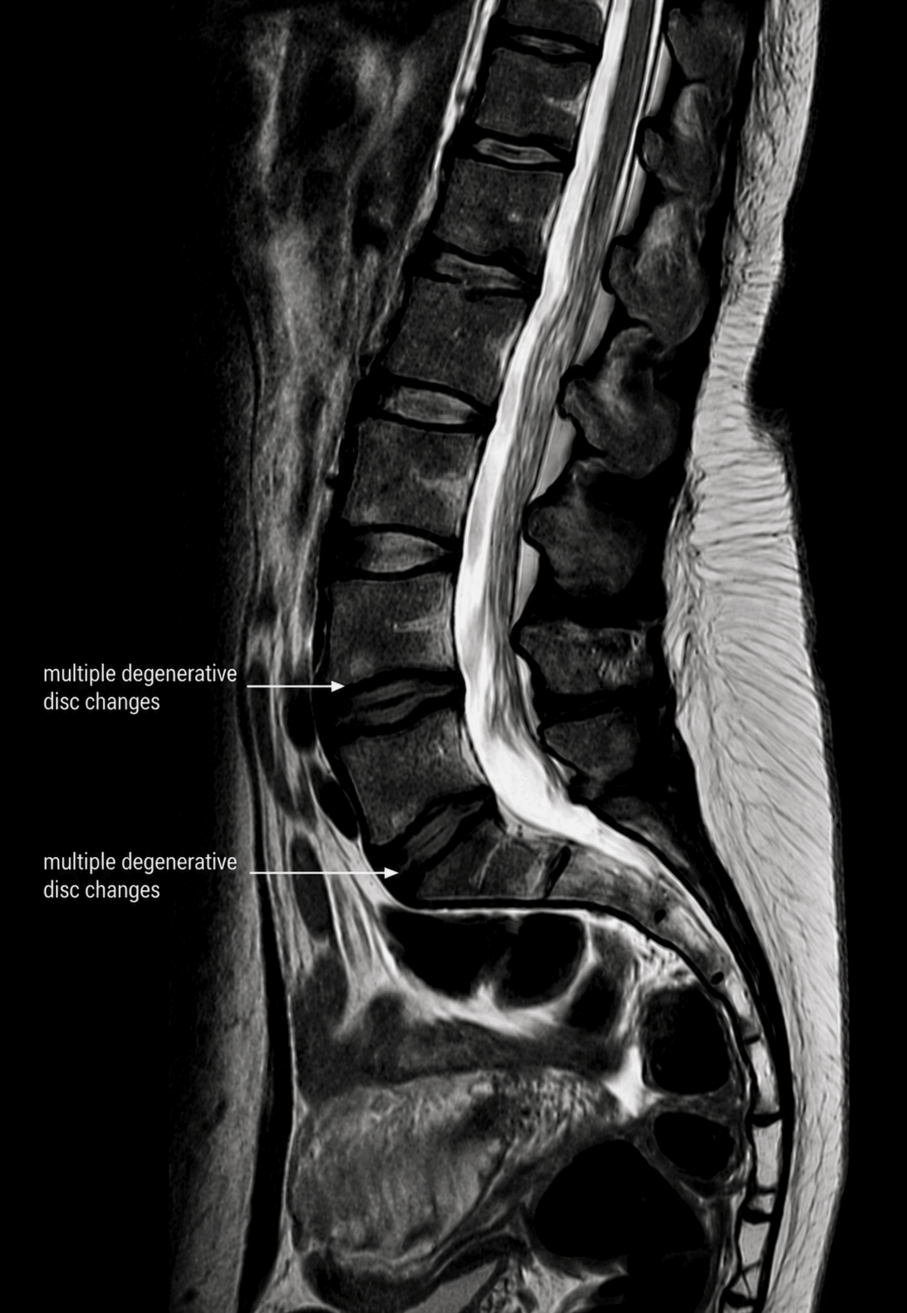
MRI of the lumbar spine MRI of the lumbar spine demonstrating mild degenerative disc changes without a compressive lesion or structural abnormality capable of explaining the recurrent neurological and autonomic manifestations.

Repeated imaging did not reveal an alternative diagnosis. CT of the abdomen and pelvis showed no acute surgical pathology, obstruction, pancreatitis, inflammatory bowel disease, gynecologic mass, or ischemic process. Findings were limited to non-specific mesenteric lymph nodes, postoperative gallbladder/gastrectomy-related changes, and a small non-obstructive renal calculus. Representative CT imaging during symptomatic episodes is shown in Figure 1. Abdominal ultrasound did not show hepatobiliary obstruction or pancreatic pathology apart from a small non-obstructive renal calculus on the left kidney (Figure 2). Transvaginal ultrasound showed polycystic ovarian morphology without adnexal mass or acute gynecologic pathology. MRI of the lumbar spine showed mild degenerative changes without a lesion capable of explaining the recurrent neurovisceral attacks (Figure 3).

Because of the severity and atypicality of the case, an extensive metabolic and toxicologic evaluation was performed. Lead level was normal. Ceruloplasmin was normal. Pyruvate and lactate were not suggestive of mitochondrial disease. The acylcarnitine profile was normal. Urine organic acid analysis showed elevated 3-hydroxybutyric acid, consistent with ketosis and catabolic stress, but did not show a pattern supportive of organic acidemia, mitochondrial dysfunction, fatty acid oxidation disorder, peroxisomal disease, or other inborn errors of metabolism. These findings supported the interpretation that ketosis was secondary to reduced intake and recurrent illness rather than the primary disease mechanism.

Serial urinary porphyrin fractionation repeatedly demonstrated abnormalities. Earlier testing showed elevated coproporphyrin I and coproporphyrin III, followed by progressive and persistent uroporphyrin elevation. Uroporphyrins reached a peak of 397 µg/L against a reference range of less than 20 µg/L, while coproporphyrin III rose as high as 275 µg/L against a reference range of less than 49 µg/L. Subsequent testing continued to show elevated uroporphyrins and coproporphyrins, demonstrating reproducible heme pathway dysregulation rather than a single isolated abnormality. Uroporphyrin elevation was particularly striking, reaching 397 µg/L (reference <20 µg/L), representing nearly 20-fold elevation above the upper reference limit and supporting substantial heme pathway dysregulation.

The major diagnostic obstacle was the repeated failure to capture elevated ALA and PBG. On review, this was attributed to delayed urine collection. During severe attacks, the patient was distressed and requested immediate treatment because she had learned from prior experience that glucose and hemin were the only interventions that improved her symptoms. Although urine collection before treatment was repeatedly requested, she often delayed sample submission for 12 to 36 hours after treatment had already begun. This likely contributed to false-negative ALA and PBG results.

During a subsequent severe attack, strict pre-treatment sampling was enforced before initiation of glucose or hemin therapy. This finally captured biochemical precursor elevation. Urinary ALA was elevated at 3.8 µmol/mmol creatinine. Later testing during an acute symptomatic episode demonstrated elevated urinary PBG, with urinary PBG 1.69 mg/L and PBG/creatinine ratio 1.02 µmol/mmol creatinine, above the reference threshold. Serial porphyria-related biochemical investigations, including progressive abnormalities in urinary porphyrin fractionation and subsequent biochemical confirmation with elevated urinary ALA and PBG obtained prior to treatment initiation, are summarized in Table 2.

**Table 2:** Porphyria-related biochemical investigations during symptomatic episodes

Test	Patient Value	Units	Reference Range	Interpretation
Urinary δ-aminolevulinic acid (ALA)	3.8	µmol/mmol creatinine	<2.6	Elevated
Urinary porphobilinogen (PBG)	1.69	mg/L	<0.89	Elevated
Urinary PBG/Creatinine ratio	1.02	µmol/mmol creatinine	<0.89	Elevated
Peak uroporphyrins	397	µg/L	<20	Markedly elevated
Coproporphyrin I	57	µg/L	0–15	Elevated
Coproporphyrin III	275	µg/L	0–49	Markedly elevated
Pentacarboxyl porphyrin	4	µg/L	0–2	Elevated

These results, interpreted in the context of recurrent neurovisceral attacks, dark urine, persistent porphyrin fraction abnormalities, and hemin responsiveness, confirmed acute hepatic porphyria. A summary of the major investigations performed during the diagnostic workup is presented in Table 3.

**Table 3:** Extensive diagnostic evaluation performed during the patient’s prolonged neurovisceral disease course

Investigation Category	Investigation	Result	Clinical Interpretation/Relevance
Structural imaging	CT of the abdomen and pelvis with contrast	No acute abdominal pathology; nonspecific mesenteric lymph nodes; small non-obstructive renal calculus; mild postoperative intrahepatic biliary prominence	No surgical, inflammatory, ischemic, pancreatic, obstructive, or structural explanation for recurrent neurovisceral attacks
	Abdominal ultrasound	No significant hepatobiliary or pancreatic pathology	Reduced likelihood of biliary obstruction, gallstone-related disease, or pancreatic inflammatory pathology
Gynecologic evaluation	Transvaginal/Pelvic ultrasound	Polycystic ovarian morphology without acute pelvic pathology	No definitive gynecologic explanation for recurrent severe attacks
Neurological imaging	MRI of the lumbar spine	Mild degenerative changes only	No compressive or structural neurological lesion explaining neuropathic symptoms
Toxicology	Blood lead level (µg/dL)	0.15 (normal range <5)	Excluded lead toxicity and lead-associated porphyria mimic
Metabolic/Hepatic evaluation	Ceruloplasmin (mg/dL)	46 (20-45)	Wilson disease unlikely
Mitochondrial evaluation	Serum lactic acid (mmol/L)	1.1 (normal range <2)	Against significant mitochondrial dysfunction or respiratory chain disorder
	Serum pyruvate (mg/dl)	0.5 (normal range 0.0-0.7)	Against pyruvate metabolism disorder or mitochondrial respiratory chain dysfunction
Fatty acid oxidation evaluation	Acylcarnitine profile	Normal tandem mass spectrometry profile	No evidence of fatty acid oxidation defect
Organic acid analysis	Urine organic acids	Elevated 3-hydroxybutyric acid only	Consistent with ketosis/catabolic stress rather than primary organic acidemia
Metabolic screening	Urine metabolic profile	No diagnostic abnormalities in amino acid, fatty acid, peroxisomal, neurotransmitter, or mitochondrial pathways	No evidence of a major inborn error of metabolism
Gastrointestinal evaluation	Fecal calprotectin	Non-inflammatory/Negative pattern	Inflammatory bowel disease less likely
	Fecal elastase	Within the acceptable range	Significant pancreatic exocrine insufficiency unlikely
Porphyria evaluation	Serial urinary porphyrin fractionation	Persistent elevation of uroporphyrins and coproporphyrin III with progressive biochemical evolution over time	Supported evolving heme biosynthesis pathway dysregulation
	Urinary δ-aminolevulinic acid (ALA)	Elevated during an acute symptomatic episode	Biochemical evidence supporting acute hepatic porphyria
	Urinary porphobilinogen (PBG)	Elevated during symptomatic attack when sampled prior to treatment	Confirmatory biochemical evidence for acute hepatic porphyria
Genetic evaluation	Whole genome sequencing (WGS)	No pathogenic acute hepatic porphyria-associated variant identified	Negative genomic testing did not exclude clinicobiochemical acute hepatic porphyria
	FECH gene variant	Likely benign/Variant of uncertain significance	Not clinically consistent with the patient’s neurovisceral phenotype

The patient was treated during severe attacks with continuous 10% dextrose infusion and intravenous hemin. Dextrose infusion consistently reduced pain intensity and improved systemic symptoms during milder or early attacks. In severe attacks with neurological and autonomic features, hemin produced more definitive improvement, including reduction of abdominal pain, improvement of paresthesias, and stabilization of autonomic symptoms. Repeated treatment courses became increasingly difficult because of hypotension during attacks and progressively difficult venous access from recurrent admissions and infusions. In one episode, hydrocortisone was administered for suspected relative adrenal insufficiency in the setting of severe systemic stress and hypotension.

Whole-genome sequencing did not identify a pathogenic variant explaining AHP. Incidental FECH variants classified as likely benign or variants of uncertain significance were reported but were not clinically relevant to the neurovisceral phenotype. The diagnosis was therefore established on clinical and biochemical grounds rather than genetics. Given the high-frequency, hemin-dependent disease course, progressive functional decline, and risk of irreversible neurological injury, givosiran therapy was pursued and approved. 

Following approval, the patient was initiated on monthly givosiran therapy and tolerated treatment without immediate adverse effects. During the initial follow-up period after initiation of therapy, she demonstrated substantial early clinical improvement, with near complete cessation of severe neurovisceral attacks. 

At approximately three months following givosiran initiation, the patient demonstrated a substantial reduction in debilitating neurovisceral attacks and a clinically meaningful improvement in pain burden. Prior to therapy, she required six admissions over four months for recurrent attacks, whereas after treatment initiation, she did not experience severe inpatient attacks thereafter. Persistent nausea, reduced appetite, and poor weight recovery remained ongoing, suggesting incomplete symptomatic resolution despite a marked reduction in attack frequency and severity. The patient nevertheless reported significant overall improvement and expressed a strong willingness to continue therapy.

## Discussion

This case illustrates the full diagnostic and therapeutic complexity of AHP in real-world practice. It is not simply a case of delayed diagnosis; it is a longitudinal demonstration of how AHP may evolve, hide biochemically, respond therapeutically before laboratory confirmation, remit during pregnancy, flare postpartum, and finally declare itself when sampling is performed at the correct moment [2,3,5,9].

Porphyrias arise from disturbances in heme biosynthesis. In the liver, heme production is tightly regulated by ALAS1, the first and rate-limiting enzyme of the pathway. ALAS1 expression is induced by fasting, stress, illness, certain medications, hormonal fluctuations, and increased hepatic cytochrome P450 demand [2,3]. In AHPs, a partial deficiency of a downstream enzyme creates a bottleneck. When ALAS1 is induced, upstream precursors accumulate. ALA and PBG are particularly important in acute attacks, with ALA considered a major neurotoxic mediator [2,3]. Importantly, AHP should not be viewed as a static binary biochemical disorder but rather as a dynamic metabolic disease with highly variable penetrance and evolving clinical expression [4,9]. Many genetically susceptible individuals remain asymptomatic or minimally symptomatic for years, while others progress from low-frequency or partially expressed attacks to severe recurrent neurovisceral disease under the influence of hormonal, metabolic, nutritional, pharmacologic, and environmental stressors [3,4]. In a subset of patients, repeated attacks may eventually transition into chronic active disease characterized by frequent hospitalizations, persistent symptoms between attacks, recurrent hemin exposure, and major impairment in functional status and quality of life [9,10]. This evolving biological behavior may explain why some patients initially demonstrate incomplete or intermittent biochemical abnormalities before developing fully expressed disease phenotypes.

The neurological manifestations of AHP are multifactorial. ALA may generate oxidative stress, impair mitochondrial function, interfere with GABAergic signaling, and disrupt axonal energy metabolism [2,11]. Autonomic nerves are particularly vulnerable, explaining tachycardia, blood pressure instability, gastrointestinal dysmotility, bladder symptoms, and severe abdominal pain in the absence of structural disease [3,5]. Peripheral motor and sensory nerves may also be affected, producing paresthesias, weakness, neuropathic pain, and, in severe cases, respiratory involvement [3,11]. Repeated attacks may also contribute to cumulative neurological injury over time. Increasing evidence suggests that patients with recurrent AHP may experience chronic symptoms even between acute attacks, including fatigue, neuropathic pain, cognitive dysfunction, anxiety, autonomic instability, and impaired quality of life [9-12]. The transition from infrequent attacks to a high-frequency hemin-dependent state in this patient may therefore reflect not only increasing metabolic instability but also progressive neurophysiological sensitization and cumulative systemic burden [10-12].

The early phase of this patient’s disease demonstrates why AHP is frequently missed. She had repeated attacks with abdominal pain, vomiting, anorexia, palpitations, tachycardia, hypotension, weakness, and fatigue. However, because the attack frequency was relatively low and initial ALA/PBG tests were negative, the diagnosis remained unconfirmed. Repeated normal imaging and intermittently negative biochemical investigations also increase the risk of patients being misclassified as having functional, psychosomatic, or unexplained chronic pain syndromes, particularly when attacks fluctuate over time [13]. This is a common diagnostic trap. AHP is often approached as a binary laboratory diagnosis, but in practice, the disease requires integration of phenotype, timing, triggers, biochemical sampling, and response to pathway-specific treatment [5,14].

A particularly important teaching point is that the patient responded to 10% dextrose and hemin before biochemical confirmation was available. Dextrose is not a conventional analgesic. Its effect in AHP is mediated through carbohydrate-dependent suppression of hepatic ALAS1, partly through insulin and glucose-responsive transcriptional pathways [2,15]. By reducing ALAS1 activity, glucose decreases flux through the heme pathway and lowers production of neurotoxic precursors [2,15]. This explains why patients with AHP may experience improvement in pain and systemic symptoms after carbohydrate loading, particularly in mild or early attacks [8,15].

Hemin acts more directly and powerfully. It provides exogenous heme, restoring negative feedback inhibition of hepatic ALAS1 [2,5]. This suppresses precursor production and can abort severe attacks [5,15]. In this case, the repeated and reproducible response to hemin, particularly improvement of neurological symptoms and autonomic instability, strongly supported the clinical diagnosis. The reproducibility and specificity of response to pathway-directed metabolic suppression strongly suggested a biologically coherent inducible heme pathway disorder rather than nonspecific analgesic responsiveness.

However, repeated hemin use is not benign. In patients with recurrent attacks, repeated hemin therapy may itself become a major source of morbidity [4,16]. Beyond thrombophlebitis and iron overload, recurrent infusions create increasing procedural complexity, venous sclerosis, difficult vascular access, hospitalization burden, psychological distress, and dependence on emergency-based care. In this patient, progressive difficulty in obtaining venous access became an increasingly important practical challenge and further reinforced the need for preventive disease-modifying therapy rather than episodic rescue management alone [15,16].

Recurrent hemin administration in severe AHP creates practical therapeutic dilemmas. Patients often require repeated peripheral cannulation, peripherally inserted central catheter (PICC) insertion, or central venous access, increasing thrombosis risk, vascular injury, catheter-associated complications, and procedural burden [16]. In patients with autonomic instability and hypotension, administration itself may become clinically challenging, particularly during recurrent admissions requiring urgent rescue therapy.

The pregnancy-related remission and postpartum deterioration are mechanistically important. Pregnancy induces complex changes in hepatic metabolism, hormonal signaling, insulin sensitivity, nutritional state, plasma volume, and heme demand [5,17]. In some women with AHP, pregnancy may stabilize attacks, possibly through altered steroid hormone dynamics, improved nutritional continuity, reduced catabolic stress, and altered hepatic ALAS1 regulation [17]. The postpartum period, in contrast, may be destabilizing. Hormonal withdrawal, sleep deprivation, lactation-related metabolic demand, reduced intake, stress, and catabolism may collectively induce ALAS1 activity [5,17]. In this patient, the postpartum period transformed a lower-frequency, partially expressed phenotype into a severe classical neurovisceral syndrome. This temporal association strongly supports the role of hormonal and metabolic modulation in disease expression [17].

The biochemical evolution of this case is another major learning point. Persistent elevation of coproporphyrin III and progressive uroporphyrin elevation signaled ongoing heme pathway disturbance before ALA/PBG confirmation was captured [4]. However, porphyrin fractionation alone is not sufficient to prove acute neurotoxic precursor accumulation because secondary porphyrinuria may occur in hepatic stress, fasting, illness, and other conditions [13]. The decisive moment came when ALA and PBG were finally obtained before treatment. This confirms the central practical lesson: in suspected AHP, urine for ALA and PBG must be collected immediately during symptoms and before glucose or hemin whenever feasible [5,13].

Although urinary ALA elevation was modest relative to some classical diagnostic thresholds reported in severe acute attacks, interpretation in this case was based on convergent clinicobiochemical evidence rather than isolated biomarker magnitude alone, including recurrent stereotyped neurovisceral attacks, marked porphyrin fraction abnormalities, pre-treatment PBG elevation, reproducible response to glucose and hemin, characteristic postpartum worsening, and subsequent early clinical stabilization following givosiran therapy.

The delay in capturing ALA and PBG was not due to the absence of disease but due to pre-analytical failure. The patient often could not or would not provide urine before treatment, and treatment was understandably started early because of severe pain. Once glucose or hemin is administered, ALAS1 suppression begins, and precursor levels may fall [15]. Samples collected 12 to 36 hours later may underestimate the biochemical peak [4,15]. This real-world scenario explains why clinicians may be falsely reassured by normal results [13].

The negative WGS result also deserves careful interpretation. AHP is commonly inherited, and pathogenic variants in HMBS, CPOX, PPOX, or ALAD can confirm subtype [1,2]. However, genetic testing does not replace biochemical diagnosis [3]. Some variants may be missed due to limitations in the detection of deep intronic, regulatory, structural, mosaic, or currently unclassified variants [18]. In addition, variant interpretation depends on existing databases and phenotype correlation. It is well established that negative WGS does not exclude AHP when clinical and biochemical criteria are fulfilled. The incidental FECH finding in this patient is not relevant to her neurovisceral phenotype, as FECH-related disease is associated with erythropoietic protoporphyria, typically presenting with photosensitivity rather than acute neurovisceral attacks [1].

This case also highlights an emerging limitation of modern genomics-driven medicine. Increasing dependence on advanced sequencing technologies may inadvertently create false diagnostic reassurance when genetic testing is negative [18]. However, clinicobiochemical syndromes with evolving metabolic expression may remain genetically unresolved because of deep intronic variants, regulatory abnormalities, modifier genes, epigenetic influences, incomplete variant databases, or currently unrecognized pathogenic mechanisms. In such disorders, longitudinal phenotype analysis remains critically important and should not be overridden by negative genomic findings alone [1,18].

This case also demonstrates the importance of longitudinal clinical acumen. The diagnosis was not made by a single test or a single admission. It required sustained recognition of a repeating pattern across years, insistence on correct sampling timing, refusal to dismiss the patient as functional despite negative early tests, and understanding that a metabolic disease may evolve before becoming biochemically obvious [5,13]. Correct diagnosis changed the therapeutic trajectory from repeated emergency care to disease modification [14].

This case highlights an important limitation of fragmented episodic medicine in rare metabolic disorders. In clinical practice, rare diseases are often missed not because diagnostic tests are unavailable, but because longitudinal biological patterns are not integrated across time, specialties, and healthcare systems [13,14]. Repeated stereotyped physiology, characteristic autonomic manifestations, trigger-response relationships, and mechanism-specific therapeutic responses may sometimes provide stronger early clues than isolated laboratory snapshots obtained under metabolically altered conditions.

Givosiran represents a major therapeutic advance in the management of recurrent acute hepatic porphyria and fundamentally changes the treatment paradigm from episodic rescue therapy to upstream molecular suppression of disease activity. It is a hepatocyte-targeted small interfering RNA (siRNA) therapeutic conjugated to N-acetylgalactosamine that selectively suppresses hepatic ALAS1 messenger RNA through RNA interference mechanisms. By reducing ALAS1 expression, givosiran decreases production of ALA and PBG, thereby directly targeting the principal upstream driver of acute neurovisceral attacks [19].

The pivotal ENVISION phase III trial demonstrated that monthly givosiran therapy significantly reduced annualized attack rates, hemin requirements, and healthcare utilization in patients with recurrent AHP. In addition to marked reductions in urinary ALA and PBG levels, the study demonstrated meaningful improvements in daily functioning, chronic pain burden, and quality of life. Importantly, ENVISION established RNA interference therapy as a true disease-modifying treatment rather than a symptomatic rescue therapy alone.

In this patient, approval and initiation of givosiran marked a major turning point in the disease trajectory. Before diagnosis and targeted therapy, she experienced years of fragmented evaluations, recurrent emergency visits, progressive nutritional decline, repeated hemin-dependent hospitalizations, and severe impairment in quality of life across multiple healthcare systems. Following recognition of the underlying metabolic disorder, management shifted toward mechanism-based preventive therapy. Givosiran was tolerated well, and ongoing follow-up will determine long-term effects on attack frequency, hospitalization burden, neurological stabilization, nutritional recovery, and overall quality of life [19,20].

The early therapeutic response in this patient was particularly striking. Prior to givosiran initiation, she had evolved into a high-frequency attack phenotype characterized by recurrent emergency visits, repeated inpatient admissions, progressive functional decline, and dependence on hemin-based rescue therapy. However, within the initial months of treatment, she demonstrated significant clinical stabilization, with no recurrent severe attacks or hospitalization requirement after initiation of therapy. Although long-term follow-up is ongoing, the rapid reduction in disease activity strongly supports the mechanistic role of ALAS1 suppression in controlling neurotoxic precursor accumulation and highlights the transformative potential of targeted RNA interference therapy in severe recurrent AHP [19,20].

The broader message of this case is that correct treatment changes everything. Before diagnosis, the patient experienced years of severe symptoms, repeated hospital visits, fragmented evaluations across countries, and treatment as nonspecific abdominal pain. Delayed diagnosis in AHP has important consequences beyond recurrent pain crises, including cumulative neuropathy, nutritional decline, psychological distress, opioid exposure, repeated radiation exposure from recurrent imaging, and healthcare utilization burden.

After recognition of AHP, care became mechanistic, targeted, and preventive. The case emphasizes that rare disease diagnosis often depends not only on access to advanced testing but also on the clinician’s ability to identify biological coherence in a long, inconsistent, and emotionally exhausting clinical course [13,14].

## Conclusions

This case demonstrates how AHP may remain clinically recognizable yet biochemically elusive for years, particularly when disease expression is evolving, and urine sampling occurs after metabolic suppression therapy. It highlights the limitations of isolated laboratory interpretation and negative genomic testing when evaluated outside the broader clinicobiochemical context. Diagnosis in this patient ultimately depended on longitudinal recognition of a highly stereotyped neurovisceral syndrome, evolving porphyrin abnormalities, reproducible response to pathway-directed therapy, and strict pre-treatment biochemical sampling during acute attacks. The dramatic postpartum escalation and subsequent stabilization following givosiran therapy further emphasize the dynamic metabolic nature of AHP and the transformative potential of mechanism-based disease-modifying treatment.

As a single-patient report, caution is warranted regarding generalizability. Although longitudinal follow-up remains ongoing and genetic sequencing did not identify a pathogenic AHP variant, diagnosis in this case was based on convergent clinicobiochemical evidence, repeated pathway-specific therapeutic responsiveness, pre-treatment biochemical confirmation, and subsequent marked reduction in attack burden following givosiran initiation. Most importantly, this case underscores that in rare metabolic diseases, biological coherence across time may be diagnostically more important than isolated negative investigations obtained at the wrong metabolic moment.
